# Performance Characterization of Recycled Carbon Fiber and Its Interfacial Bonding Properties with Cement Matrix

**DOI:** 10.3390/ma18071532

**Published:** 2025-03-28

**Authors:** Tao Shi, Kunming Li, Sijie Shao, Xuanfeng Cai, Xinpeng Wang, Chonggen Pan, Ning Fu, Haibo Wang

**Affiliations:** 1 College of Civil Engineering, Zhejiang University of Technology, Hangzhou 310023, China; 1112106010@zjut.edu.cn (K.L.); 211122060028@zjut.edu.cn (S.S.); 221122060138@zjut.edu.cn (X.C.); 221123060143@zjut.edu.cn (X.W.); 2 Zhejiang Key Laboratory of Civil Engineering Structures & Disaster Prevention and Mitigation Technology, Zhejiang University of Technology, Hangzhou 310023, China; 3 School of Civil Engineering, NingboTech University, Ningbo 315100, China; panchonggen2000@163.com; 4 Ningbo Jiaochuan Component Co., Ltd., Ningbo 315207, China; funing197@163.com (N.F.); wanghaibo145@163.com (H.W.)

**Keywords:** recycled carbon fiber, single-fiber pull-out, interfacial bonding properties, bridging stress

## Abstract

Recycled carbon fiber, as a novel type of solid waste, possesses high tensile strength, structural stability, and low utilization rates. Recycling carbon fiber for use in cementitious materials presents an efficient solution. However, achieving good interfacial bonding between recycled carbon fiber and cementitious materials is crucial for its high-performance application in such materials. This study first characterizes the properties of recycled carbon fiber and, for the first time, tests the interfacial parameters between recycled carbon fiber and cement matrix through single-fiber pull-out tests. The results show that the surface of recycled carbon fiber, lacking active functional groups and being relatively smooth, leads to poorer interfacial bonding with the cement matrix compared to virgin carbon fiber. The interfacial bonding strength, interfacial friction bonding strength, and chemical debonding energy are 0.65 MPa, 0.47 MPa, and 0.36 J/m^2^, respectively. Next, based on the theoretical model of interfacial mechanics, a single-fiber pull-out model was used to predict the bridging stress curve of recycled carbon fiber. The calculations show that the bridging stress of recycled carbon fiber at volume fractions of 0.16%, 0.3%, and 0.47% are 1.25 MPa, 2.18 MPa, and 3.40 MPa, respectively. Finally, tensile tests were conducted to investigate the tensile properties of cementitious materials reinforced with recycled carbon fiber. At various fiber contents, the recycled carbon fibers provided corresponding bridging stresses at crack sites, enhancing the tensile strength of the cementitious materials by 8.8~35.48%.

## 1. Introduction

Carbon fiber is a high-performance reinforcing material [1,2] and is typically processed into carbon fiber-reinforced polymer (CFRP) in industry applications. The lightweight, high-strength properties of CFRP have made it highly sought after across various sectors [3,4,5,6]. However, with the rapid growth in demand for CFRP, there has been a dramatic increase in carbon fiber waste accumulation [7]. It is projected that by 2050, the global generation of CFRP waste in the wind, energy, and aerospace sectors will reach 43.4 million and 500,000 tons, respectively [8,9]. Carbon fiber production, however, is energy intensive, requiring approximately 100–600 MJ of energy to produce 1 kg, and costs up to 45 USD per kilogram [10,11,12]. Unfortunately, current disposal methods for CFRP waste primarily involve landfilling and incineration, leading to increased environmental pressure and significant resource waste. Extensive research has shown that recycled carbon fiber (RCF) exhibits performance comparable to virgin carbon fiber (VCF), with tensile strength reaching up to 80% of that of the original material [13,14], while costing as low as 15% of VCF’s price [15]. Therefore, the recycling and reuse of RCF offers both environmental and economic benefits. Currently, the main methods for recycling waste carbon fibers include mechanical recycling, thermal recovery, and chemical recovery.

Cementitious materials often face issues such as susceptibility to cracking, low toughness, and high brittleness during service. To address these problems, researchers typically incorporate fibers into cementitious materials for structural modification [16]. In fiber-reinforced cementitious materials, the interface between the fibers and the matrix serves as a bridge for load transfer, where stress is transferred from the matrix to the fibers through the interface, which is responsible for the reinforcement and toughening effects. Therefore, understanding the bonding performance between the fibers and the matrix is crucial. The single-fiber pull-out test, due to its relatively low equipment requirements, reliable results, and ability to directly obtain the load–displacement curve of a single fiber being pulled out from the matrix along with numerous interfacial parameters, has been widely applied in studying fiber–matrix interfacial performance. It is an important characterization method. In early studies, Redon et al. [17] used the single-fiber pull-out test to measure the bonding performance of polyethylene vinyl alcohol (PVA) fibers with different diameters in mortar. The results indicated that the chemical bonding between PVA and mortar was relatively strong, with the chemical debonding energy ranging from 4 to 5 J/m^2^. Additionally, the single-fiber pull-out test demonstrated that even if fiber breakage occurred during the pull-out process, the interfacial bond strength could still be determined, as long as complete fiber debonding could be independently verified. Wu et al. [18] employed scanning electron microscopy (SEM) combined with the single-fiber pull-out test to study the failure mechanisms of carbon fiber-reinforced cement. The results showed that the increase in interfacial strain was accompanied by fiber pull-out, fiber breakage, and interfacial debonding, providing a basis for numerical simulation analysis. Currently, researchers focus their studies on the interface bonding properties between fibers and the matrix, particularly for steel fibers, polyvinyl alcohol (PVA) fibers, and polyethylene (PE) fibers [19,20,21], and based on this, a series of methods for enhancing and modifying the interface bonding performance of these fibers have been proposed.

Steel fibers can be made into various shapes, such as deformed steel fibers, hooked-end steel fibers, and corrugated steel fibers, to enhance the anchoring effect [22,23]. Wu et al. [24] found that corrugated and hooked-end steel fibers showed significantly improved fiber–matrix interface bonding performance and macroscopic mechanical properties compared to straight steel fibers. This improvement is attributed to the increased interface friction between the fibers and the matrix. For synthetic fibers like PVA and PE, the surface modification or grafting of chemical functional groups is commonly used to enhance the interface bonding [25]. Meng et al. [26] prepared PVA-reinforced cementitious materials by modifying PVA fibers with nanosilica. Their research showed that the flexural strength and ultimate deflection of nanosilica-modified PVA fiber-reinforced cement composites increased by 28% and 74%, respectively, compared to unmodified fiber-reinforced cementitious materials. He et al. [27] enhanced the interface friction stress between fibers and the cement matrix by coating PE fibers with carbon nanofibers through hydrophobic interactions. This reinforced the interface transition zone. As a result, the composite material’s interface friction strength increased by 22%, improving its tensile properties.

Although researchers have gradually begun studying recycled carbon fiber (RCF) reinforced cementitious materials, offering new ideas and directions for the green recycling of carbon fiber waste, current research mainly focuses on the basic mechanical properties, such as compressive and flexural strength, as well as electrical conductivity [28,29]. It is noteworthy that extensive research on the carbon fiber–polymer interface has demonstrated that surface modification techniques—such as oxidation etching [30], vapor deposition [31], or chemical grafting [32]—can significantly enhance interfacial adhesion by improving chemical bonding and mechanical interlocking between the fibers and polymer matrix. However, there remains a significant research gap regarding how these well-established surface treatment techniques can be applied to cement matrices, particularly in the case of recycled carbon fibers, which may experience performance degradation during the recycling process. Moreover, existing studies lack systematic investigations on the interface bonding performance between RCF and the cement matrix. Furthermore, the fundamental role of fibers in reinforcing cementitious materials lies in their ability to provide bridging stress at the cracks of the matrix. However, no studies have been conducted on the bridging stress of RCF so far. Based on this, this study, for the first time, tests the interface bonding parameters between recycled carbon fibers and the cement matrix using a single-fiber pull-out test and derives the RCF bridging stress curve based on a micro-scale pull-out model for a single fiber. At the same time, through macro tensile tests, the reinforcing mechanism of RCF in cementitious materials is explored. Additionally, by comparing the performance characterization tests of RCF and virgin carbon fiber (VCF), the reasons for their differences in macroscopic performance enhancement and interface bonding are discussed. This study aims to lay a theoretical foundation for the surface modification of RCF to improve overall performance and for future engineering applications.

## 2. Experimental Materials and Methods

### 2.1. Materials

The materials used in this study include cement, water, fine aggregate, a high-efficiency water-reducing agent, recycled carbon fibers, and virgin carbon fibers. The cement is ordinary Portland cement of grade 42.5, with the relevant physical properties listed in Table 1. The fine aggregate is river sand with low clay content and good gradation, with a fineness modulus of 2.7. Tap water and a polycarboxylate high-efficiency water-reducing agent with a water reduction rate of approximately 30% were used. The RCFs were sourced from yarn waste produced by carbon fiber manufacturers, with mechanical cutting as the recycling method. The virgin carbon fibers were purchased from Hangzhou Gaoke Composite Materials Co., Ltd. (Hangzhou, China). Their performance parameters are shown in Table 2.

### 2.2. Tensile Performance Testing of RCF-Reinforced Cementitious Materials

**Figure 1:**
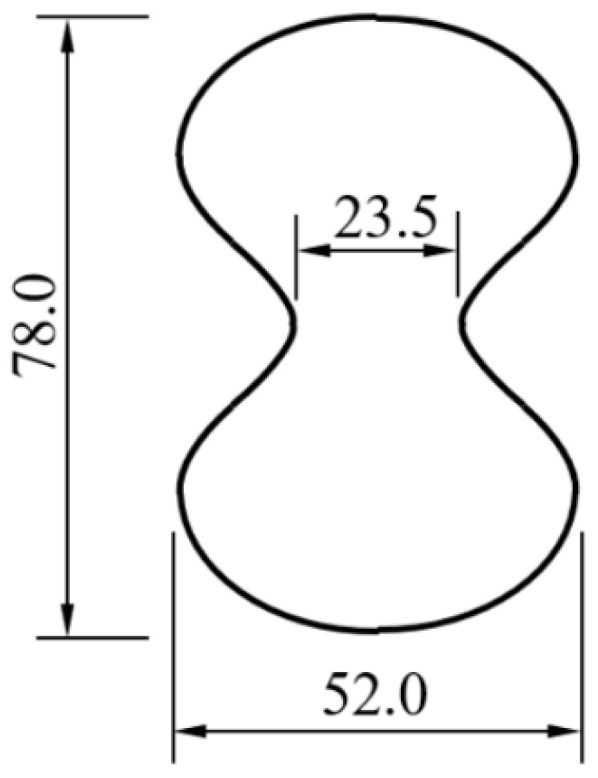
Shape-stretching mold diagram (unit: mm).

To investigate the tensile properties of RCF-reinforced cementitious materials, this study considered three fiber volume fractions (0.16%, 0.3%, and 0.47%) for two types of carbon fibers. The fiber volume fraction was calculated based on the fiber content as a percentage of cement mass. Considering the high water absorption rates of RCF and VCF, the fiber content should not be too high, as this would lead to a decline in the workability of the cement-based material, resulting in a less dense matrix and thus reduced mechanical properties. Table 3 presents the proportions of the mix for seven different mixtures, with three specimens prepared for each mix. The mixtures were poured into “8”-shaped molds with cross-sectional dimensions of 22.5 mm × 52 mm. The mold morphology used to test the tensile strength of cement-based materials reinforced with RCF or VCF is shown in Figure 1. During specimen preparation, the flowability of the different mix proportions was tested, and the results are provided in Table 3. Here, CF0 refers to specimens without any carbon fibers, and the numbers following RCF and VCF represent the fiber volume fraction of recycled carbon fibers and virgin carbon fibers, respectively.

**Figure 2:**
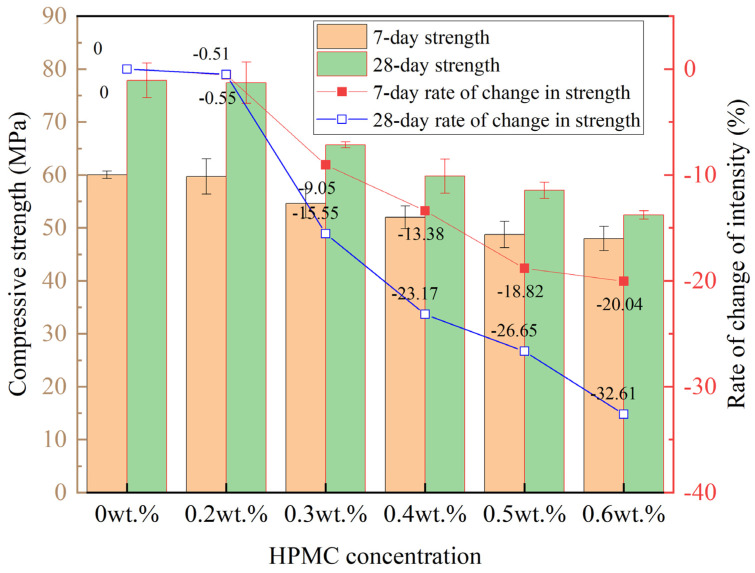
Effect of different HPMC dosages on the compressive strength of RCF-reinforced cement paste.

Achieving the uniform dispersion of RCF in cementitious materials is essential to fully utilizing its reinforcing effect. Before conducting tensile strength tests, a preliminary experiment was carried out to evaluate the dispersion performance of RCF. This study first compared three dispersion methods, including ultrasonic dispersion, magnetic stirring, and hydroxypropyl methylcellulose (HPMC) dispersant combined with magnetic stirring, in terms of their effectiveness in dispersing RCF in water. The results showed that the combination of HPMC and magnetic stirring achieved the best dispersion performance. The molecular structure of HPMC contains both hydrophilic hydroxyl and hydrophobic alkyl groups. Its polar functional groups, such as the hydroxyl and carbonyl groups, can form hydrogen bonds with water molecules, leading to the formation of monomolecular layers and micellar aggregates at the liquid interface and within the solution. This process effectively reduces the contact area between water and air, resulting in a significant decrease in surface tension. As a result, HPMC enhances the hydrophilicity and wettability of RCF, promotes its uniform distribution, and ultimately forms a colloidal dispersion system [33]. Despite its excellent dispersion performance, HPMC significantly reduces the compressive strength of cementitious materials, as shown in Figure 2. This reduction is mainly attributed to the air-entraining effect of HPMC, which introduces a large number of pores into the paste. The increase in porosity negatively affects the compressive strength of cement paste. In addition, HPMC has a retarding effect that slows down cement hydration and reduces the overall degree of hydration. Consequently, the negative effects of HPMC outweigh the positive reinforcement provided by RCF, leading to a substantial decrease in specimen strength. These findings suggest that although conventional dispersion methods may not completely eliminate the potential agglomeration of RCF, they can still contribute to improving the performance of cementitious materials.

### 2.3. Fiber Performance Characterization Tests

To better understand the differences in the influence mechanisms of RCF and VCF on cementitious materials, as well as the performance differences between the two fibers, it is crucial to perform characterization tests on the fibers themselves. In this study, Fourier transform infrared spectroscopy (FTIR), atomic force microscopy (AFM) surface roughness testing, and scanning electron microscopy (SEM) tests were conducted on both RCF and VCF.

#### 2.3.1. Fourier Transform Infrared Spectroscopy (FTIR) Test

The FTIR test aims to investigate the surface oxidation functional groups of the two types of carbon fibers. The types and quantities of oxidation functional groups influence the chemical bonding performance between the carbon fibers and the cement matrix. Moreover, the varying amounts of hydrophilic functional groups affect the water absorption rate of RCF and VCF. Therefore, studying the surface oxidation functional groups of the two carbon fibers is crucial for understanding their bonding mechanisms at the interface with the cement matrix, as well as their impact on the flowability of cementitious materials. This investigation helps elucidate how these fibers influence the overall performance of cementitious composites.

#### 2.3.2. Atomic Force Microscopy (AFM) Surface Roughness Testing

The surface roughness of fibers is one of the key factors influencing the interfacial bonding strength between the fibers and the matrix. A smooth fiber surface tends to lead to slippage when combined with the cement interface. In contrast, a rougher fiber surface enhances mechanical friction between the fiber and the cement matrix, resulting in a stronger interfacial bond [34]. To explore the reasons behind the differences in the toughness enhancement effects of the two types of carbon fibers on cementitious materials, this study employed atomic force microscopy (AFM), specifically the Bruker Dimension ICON model, to scan a 3 µm × 3 µm area of the two types of carbon fibers. AFM works by scanning the sample surface and measuring atomic-level interaction forces, thereby providing detailed microstructural information about the surface morphology. The AFM 3D topography map provides an intuitive visualization of the height distribution, roughness, texture, and other microscopic structures of the sample surface. This technique allows for a precise comparison of the surface characteristics of the two carbon fibers, helping to explain their different effects on the toughness and performance of cement-based composites.

#### 2.3.3. Scanning Electron Microscopy (SEM) Testing

Scanning electron microscopy (SEM) testing can observe the microscopic morphology of RCF and VCF through high-resolution imaging technology. The trimmed carbon fibers are adhered to the sample holder using a gold-coated conductive adhesive and then placed into the sample chamber, followed by vacuuming for testing.

### 2.4. Single-Fiber Pull-Out Test

**Figure 3:**
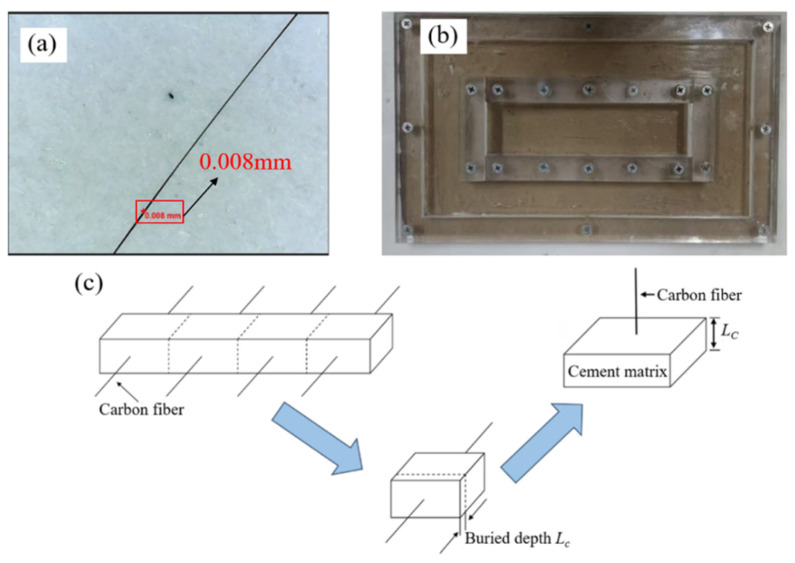
(**a**) Single-carbon-fiber diagram; (**b**) single-fiber pull-out specimen casting mold diagram; (**c**) schematic diagram of cutting process of single-fiber extraction specimen.

The characterization of the fiber–cement matrix interfacial performance is commonly conducted using fiber pull-out tests, which can be further divided into bundle pull-out and single-fiber pull-out tests. Bundle pull-out tests are relatively easier to perform; however, due to the gradual failure of the fibers from the “sleeve layer” along the “core layer”, multiple influencing factors come into play, making it less straightforward to reveal the interfacial performance between fibers and the cement matrix [35]. In contrast, single-fiber pull-out tests provide a more direct and clear insight into the interfacial performance between the fiber and the cement matrix. Single-fiber pull-out tests have now been widely used to evaluate the interfacial bonding performance between fibers and cement matrices. By conducting this test, it is possible to explore the different mechanisms by which VCF and RCF affect the toughness of cementitious materials. However, factors such as the small diameter and brittleness of a single RCF make the pull-out test operation more challenging. To address these challenges, the single-fiber pull-out tests in this study were performed according to the following procedure. Figure 3a–c illustrate the schematic of the procedure for preparing single-fiber pull-out specimens.

#### 2.4.1. Selection of a Single Carbon Fiber

This section describes the process of separating individual fibers from fiber bundles composed of multiple recycled carbon fibers. As mentioned earlier, the diameter of a single carbon fiber is about 7 μm, making it difficult to observe clearly with the naked eye. In this study, when selecting a single fiber, the carbon fiber bundle is first split into individual fibers as much as possible, and the possible single fibers are placed on a white A4 sheet. A handheld electron microscope is then used to observe them and determine if they are single fibers. If the diameter is close to the true diameter of a single carbon fiber, the fiber is set aside for future use. A single carbon fiber under the electron microscope is shown in Figure 3a.

#### 2.4.2. Mixing Ratio and Casting Process

To reduce the impact of fine aggregates, the cement paste was directly used as the base material in this experiment. Additionally, to prevent the carbon fibers from bending or breaking during the cement casting process, the cement paste needs to reach a self-leveling state. The water-to-cement ratio was set at 0.5, and 0.07 wt% of a polycarboxylate superplasticizer was added. Since a single carbon fiber is very fine and brittle, it cannot be securely embedded into the cement matrix using conventional molds. To address this issue, a custom acrylic mold was used for casting the specimens. A photo of the mold is shown in Figure 3b. After the specimens were cast, they were left undisturbed for 24 h before demolding. The specimens were carefully removed and cured for 28 days. The depth of fiber embedding into the matrix was controlled by cutting the specimens. The cutting method used was wire cutting, which provides a smooth cutting surface for cement slices. A schematic of the cutting process is shown in Figure 3c, with cutting conducted along the dashed line. The specimens were first cut into several large blocks according to the fiber arrangement direction, and then these large blocks were cut along the vertical direction of the fibers to produce small cement matrix pieces with fibers at their free ends. After cutting, the depth of fiber embedding was measured and recorded as *L_c_*.

#### 2.4.3. Determination of Critical Embedding Depth

To ensure that a single fiber can be successfully pulled out from the cement matrix without breaking, the critical embedding depth of the fiber needs to be determined before the experiment. This can be calculated using Equation (1) [36], which is derived from the shear-lag model for an isolated, perfectly aligned single fiber under the following assumptions: (1) uniform interfacial shear stress distribution along the fiber–matrix interface; (2) no fiber curvature or misalignment relative to the loading direction; (3) absence of neighboring fiber interactions (valid only for single-fiber systems).

(1) $$L_{b}=\frac{\sigma_{f}d}{4\tau_{set}}$$

where *L_b_* is the critical embedding depth of a single fiber (in mm); *σ_f_* is the tensile strength of a single carbon fiber (in MPa); *d* is the diameter of a single carbon fiber, taken as 0.007 mm; and *τ_set_* is the estimated interface bonding strength between the carbon fiber and the matrix (in MPa). According to Li et al. [36], through single-fiber pull-out tests under a water-to-cement ratio of 0.5, the interface bonding strength between carbon fiber and cement matrix was found to be 0.66 MPa. In this study, with a water-to-cement ratio of 0.5, *τ_set_* is taken as 0.66 MPa. Using Equation (2), the critical embedding depths *L_b_* for VCF and RCF were calculated to be 9.1 mm and 7.6 mm, respectively. In practice, to successfully obtain the full load–displacement curve during the pull-out process, the actual embedding depth of the carbon fiber should be further restricted. The longer the embedding depth and total length of the carbon fiber, the greater the elastic energy stored in the fiber. When the interface fails under tensile stress, the tensile force will drop suddenly, and the stored elastic energy will be released instantaneously. This may lead to a sudden pull-out and increase the risk of fiber breakage [37]. Therefore, in this study, the cutting dimensions of the VCF and RCF specimens were taken as 3 mm to control the actual embedding depth of the fibers to approximately 3 mm. After measuring the specimen to the required dimensions, marks were made, and the specimen was cut according to these marks using high-precision wire cutting to control the fiber embedding depth.

#### 2.4.4. Specimen Loading

**Figure 4:**
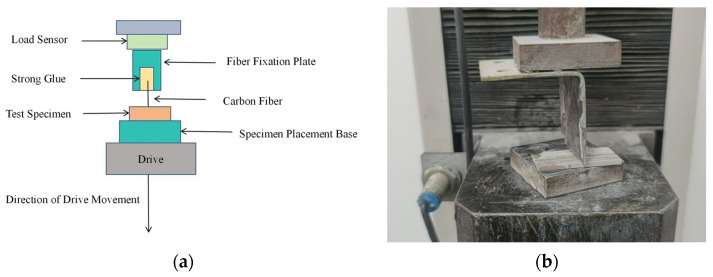
Single-fiber pull-out loading process. (**a**) Test setup diagram. (**b**) Experimental physical diagram.

The single-fiber pull-out loading process is shown in Figure 4. The test loading is performed using a universal testing machine at a rate of 0.2 mm/min. Since the load on a single fiber being pulled out is very small, a high-sensitivity, high-precision load sensor is required. The procedure involves fixing the test specimen to the base using adhesive, rotating the specimen base to adjust the specimen’s position so that it is close to the fiber fixing plate, and carefully attaching the free end of the fiber to the fiber fixing plate with adhesive. After the preparation, the universal testing machine is activated for the pull-out process, and five valid data points are taken for each group of tests.

## 3. Results and Discussion

In this study, the fiber performance characterization and interface bonding performance focus on single fibers. The interface bonding performance is analyzed based on single-fiber results to eliminate the sleeve effect caused by the complex nature of fiber bundles. Although recycled carbon fibers typically exist in bundles, isolating individual fibers allows for the precise quantification of interface parameters and provides a clearer insight into the interface performance between the fibers and the cement matrix, avoiding the interference from interactions between fibers within the bundles.

### 3.1. Tensile Strength of Recycled Carbon Fiber-Reinforced Cementitious Materials

**Figure 5:**
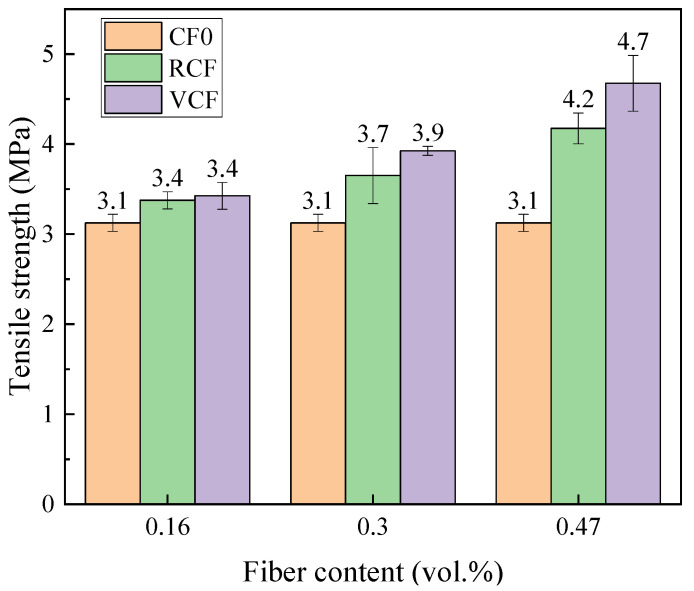
Twenty-eight-day tensile strength of different carbon fiber specimens.

In engineering structural design, although cementitious materials primarily withstand compressive stress, tensile regions inevitably arise in practical situations, especially when components are subjected to bending or shear forces. The tensile strength directly impacts the durability and safety of the structure. Figure 5 shows the 28-day tensile strength of cementitious materials reinforced with RCF and VCF. As shown in Figure 5, RCF significantly enhances tensile strength, with an increase corresponding to higher fiber content. RCF improves the tensile strength of the specimens by 8.80% to 35.48%. Cementitious materials are brittle, and when subjected to tensile forces, internal microcracks tend to propagate, leading to rapid material failure. The incorporation of RCF effectively suppresses crack propagation. When cracks form near the fibers, the fibers bridge the cracks, delaying their growth, and thereby improving the material’s toughness and tensile strength. Additionally, specimens with VCF show higher tensile strength, with an increase ranging from 12.90% to 51.60%. The difference in the strengthening effect between VCF and RCF on tensile strength is discussed in the following sections.

### 3.2. Fiber Characterization Tests

#### 3.2.1. Surface Oxidation Functional Group

**Figure 6:**
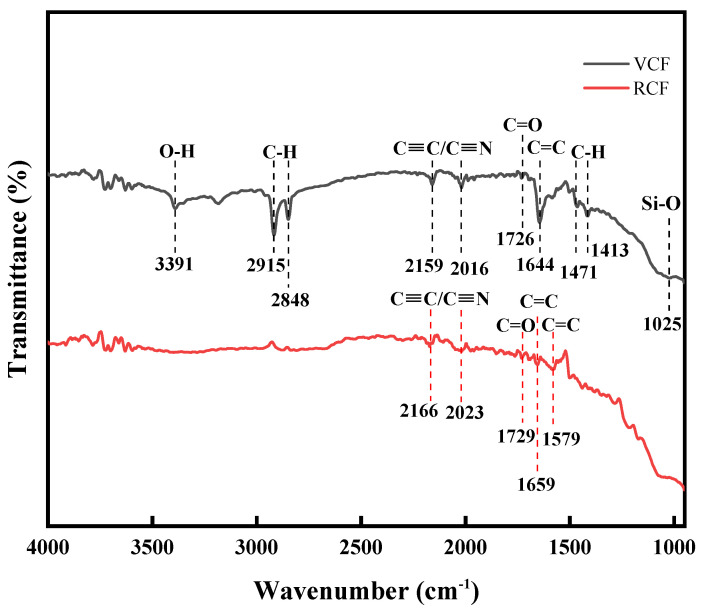
FTIR graph of different carbon fibers.

Figure 6 illustrates the infrared spectra of RCF and VCF. For RCF, stretching vibration peaks associated with alkynes (C≡C) or nitriles (C≡N) appear at 2166 cm^−^^1^ and 2023 cm^−^^1^, suggesting the presence of small amounts of conjugated alkyne (C≡C) or nitrile (C≡N) groups on the carbon fiber surface, typically introduced during high-temperature processing [38]. A stretching vibration peak of the carbonyl group (C=O) from carboxylic acid appears at 1729 cm^−^^1^ [39,40], while the absorption peaks at 1659 cm^−^^1^ and 1579 cm^−^^1^ correspond to the stretching vibrations of C=C bonds in the aromatic ring structure of the carbon fiber [41].

Comparing the infrared spectrum of VCF, in addition to the functional groups identified in RCF, VCF displays additional functional groups. The peak at 3391 cm^−^^1^ corresponds to the stretching vibration of the hydroxyl group (O-H), indicating the presence of hydroxyl groups on the VCF surface [42,43]. The peaks at 2915 cm^−^^1^ and 2848 cm^−^^1^ are attributed to the symmetric and asymmetric stretching vibrations of the alkyl (-CH_2_-) groups [44], while the peaks at 1471 cm^−^^1^ and 1413 cm^−^^1^ correspond to the bending vibrations of the C-H bonds [42]. Furthermore, a stretching vibration peak at 1025 cm^−^^1^ is observed, which is associated with Si-O bonds [40,45]. The presence of alkyl and Si-O groups suggests that the VCF surface contains silane coupling agents.

During the production process, carbon fibers undergo a pre-oxidation stage, which enables the formation of acidic functional groups on the fiber surface, such as -OH and -COOH [46,47,48]. According to the experimental results in Figure 5, due to differences in the production process, the hydroxyl content in VCF is higher than that in RCF. This may be because, during the oxidation treatment, VCF undergoes a longer treatment time, and the concentration of the oxidizing medium and the treatment temperature are slightly higher than those of RCF. Hydroxyl groups, as active functional groups, not only significantly enhance the surface energy and roughness of the fibers but also improve the interfacial chemical bonding strength and interfacial chemical debonding energy between the fibers and the cement matrix. This, in turn, increases the energy barrier that must be overcome during fiber pull-out, thereby improving the mechanical properties of the cementitious materials [49]. Additionally, relevant studies have shown that silane coupling agents can enhance mechanical friction by increasing the surface roughness of the fibers, thereby improving the interfacial bonding between the fibers and the matrix [34]. Therefore, the analysis of surface functional groups in RCF and VCF can reveal the differences in the interfacial bonding performance between the two types of carbon fibers and the cement matrix and explain why VCF has a superior effect on enhancing the tensile properties of cementitious materials compared to RCF.

#### 3.2.2. AFM Surface Roughness

**Figure 7:**
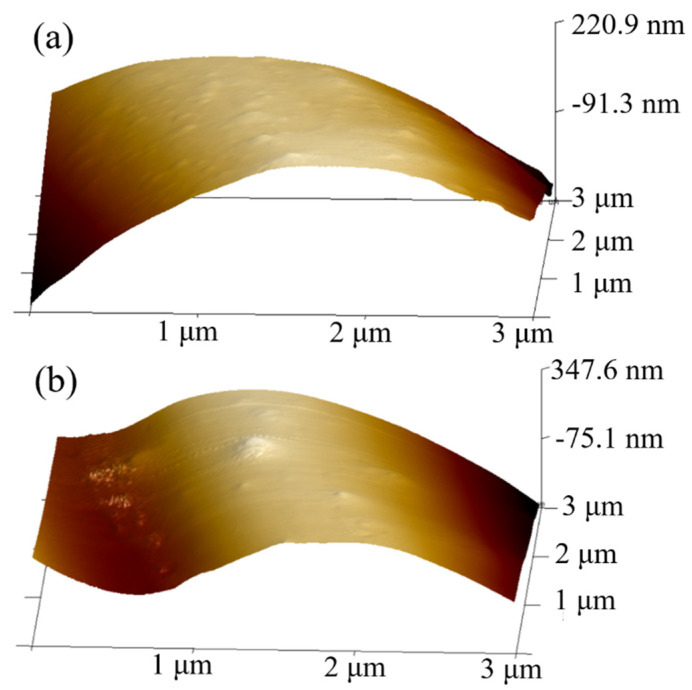
Three-dimensional morphology of two carbon fiber AFMs ((**a**) for RCF and (**b**) for VCF).

Figure 7a,b show the 3D surface morphology of RCF and VCF as observed through AFM. According to the Nanoscope analysis software v3.0, the root mean square (RMS) surface roughness and average surface roughness of RCF are 89 nm and 76.5 nm, respectively, while those of VCF are 135 nm and 119 nm. RMS surface roughness measures the “fluctuation” in surface height, typically reflecting the finer details of surface roughness. The higher the RMS surface roughness value, the greater the surface fluctuation, and the rougher the surface. Average surface roughness reflects the degree of deviation from the average surface height, considering the overall “average” surface fluctuation. A higher average surface roughness value indicates a greater variation in surface height. The surface roughness of carbon fibers is influenced by their carbonization process and surface modifiers. The greater surface roughness of VCF may be due to its higher carbonization temperature compared to RCF, as well as the silane coupling agents on the VCF surface, which further increase its roughness [50]. Therefore, VCF has a higher surface roughness, which can provide more mechanical friction. As a result, the interfacial bonding strength between VCF and the cement matrix is also higher, leading to enhanced tensile strength in cementitious materials reinforced with VCF.

#### 3.2.3. Microscopic Topography

**Figure 8:**
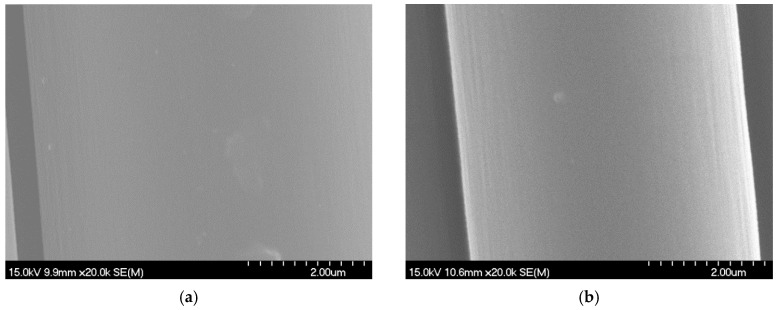
Microscopic morphology of recycled carbon fiber and virgin carbon fiber. (**a**) RCF. (**b**) VCF.

Figure 8 shows the microstructure of RCF and VCF at 20,000× magnification under scanning electron microscopy. From the image, it can be observed that both RCF and VCF surfaces are relatively smooth, with only a small number of particles attached. This may be due to the sizing treatment applied to the precursor fibers during the carbon fiber production process, which helps prevent the formation of fuzz. Additionally, since recycled carbon fiber is obtained through mechanical cutting, its surface does not exhibit the grooves and furrows typically found on the surface of pyrolyzed recycled carbon fibers. Furthermore, a careful comparison of their microstructures reveals that the surface of virgin carbon fibers has more grooves than recycled carbon fibers, with the surface of recycled carbon fibers being smoother compared to that of virgin carbon fibers.

### 3.3. Interfacial Adhesion Properties

**Figure 9:**
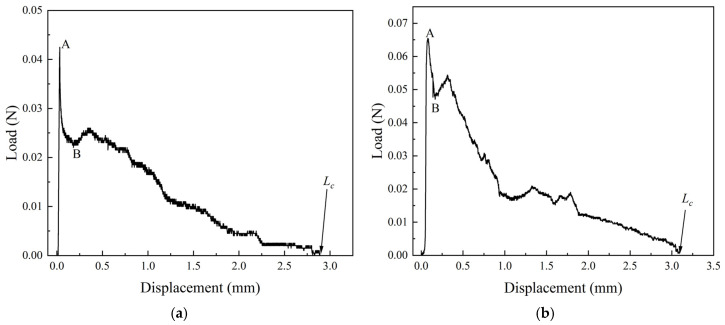
Load–displacement curves for RCF and VCF single-fiber pull-out. (**a**) RCF. (**b**) VCF.

The typical successful single-fiber pull-out load–displacement curves for RCF and VCF specimens are shown in Figure 9. The fiber pull-out process can be divided into three main stages: elastic elongation, debonding, and slippage. In the first stage, the carbon fiber undergoes elastic stretching under tensile force before complete debonding occurs. As the displacement increases, the pull-out load increases accordingly. The process then enters the debonding stage, where the load continues to rise until it reaches the peak load, Pa, at point A. Afterward, the load drops from *P_a_* to *P_b_* at point B, marking the transition from partial debonding to complete debonding. If the load suddenly drops sharply, it indicates the rupture of the chemical bond between the fiber and the matrix. Once the fiber is completely debonded, it entirely separates from the matrix, and slippage occurs inside the matrix, entering the slippage stage. In the slippage stage, the pull-out force is primarily provided by the friction at the interface. As the interface continuously breaks down, the load decreases gradually until it reaches zero. The pull-out load–displacement curve obtained in this experiment closely matches the curves reported in the literature. *L_c_* in the figure is the depth of the fiber buried in the matrix.

In the single-fiber pull-out load–displacement curve, the peak load *P_a_* corresponds to the interfacial bonding strength, which can be calculated using Equation (2) [51].

(2) $$\tau =\frac{P_{a}}{\pi dL_{c}}$$

where *τ* is the fiber–matrix interfacial bonding strength in MPa; *d* is the diameter of the carbon fiber in mm; *L_c_* is the depth of fiber embedment in the matrix in mm; *P_a_* is the peak load during single-fiber pull-out in N.

Starting from point *P_b_*, the fiber is primarily influenced by the frictional force at the interface. Subsequently, the chemical debonding transitions to frictional bonding. The load *P_b_* corresponds to the interfacial friction bonding strength, which can be calculated using Equation (3) [51].

(3) $$\tau_{f}=\frac{P_{b}}{\pi dL_{c}}$$

where *τ_f_* is the interfacial friction bonding strength in MPa; *P_b_* is the load at the beginning of frictional slippage after complete debonding during the single-fiber pull-out process in N.

The sharp drop in load during the transition from partial debonding to complete debonding in the fiber pull-out process indicates the presence of chemical bonding between the fiber and the cement matrix. The debonding process can be considered as crack propagation along the fiber–matrix interface, requiring the overcoming of the chemical debonding energy, *G_d_*, at the fiber–matrix interface. The chemical debonding energy can be calculated using Equation (4) [51].

(4) $$G_{d}=\frac{2{\left(P_{a}-P_{b}\right)}^{2}}{\pi^{2}E_{f}d^{3}}$$

where *G_d_* is the chemical debonding energy between the fiber and the matrix in J/m^2^; *E_f_* is the fiber’s elastic modulus in MPa.

Pull-out energy refers to the energy required to pull a fiber out of the matrix in a single-fiber pull-out test. It is typically defined as the area enclosed by the load–displacement curve for a single-fiber pull-out and the corresponding coordinate axes. The pull-out energy value is usually calculated by integrating the experimental data (force–displacement curve) and can be calculated using Equation (5).

(5) $$W_{P}=\int_{0}^{L_{c}}P\left(S\right)dS$$

where *W_P_* is the fiber pull-out energy, and *P*(*S*) is the load–displacement curve for a single-fiber pull-out.

**Figure 10:**
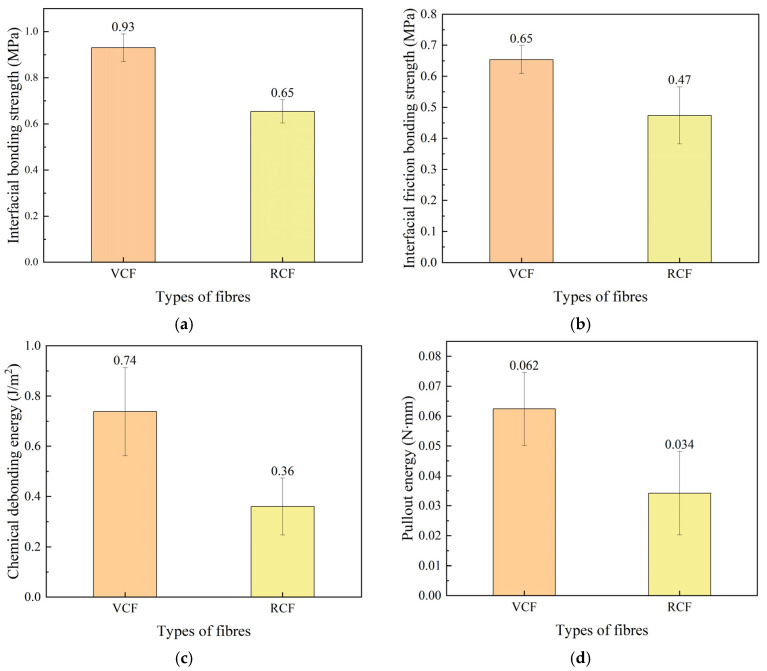
Interfacial bonding performance parameters of RCF and VCF with cement matrix. (**a**) Interfacial bonding strength. (**b**) Interfacial friction bonding strength. (**c**) Chemical debonding energy. (**d**) Pull-out energy.

Figure 10 shows the different performance parameters between RCF and VCF and the cement matrix. It can be seen from the figure that all interfacial bonding performance parameters of VCF are higher than those of RCF. Specifically, the *τ*, *τ_f_*, *G_d_*, and *W_P_* values for RCF are 0.65 MPa, 0.47 MPa, 0.36 J/m^2^, and 0.034 N·mm, respectively. Compared to RCF, the *τ*, *τ_f_*, *G_d_*, and *W_P_* values for VCF are higher by 43.1%, 38.3%, 105.6%, and 82.4%, respectively.

The interfacial interaction between carbon fibers and the cement matrix is a typical composite material interfacial bonding strength, mainly comprising physical bonding and chemical bonding [52]. Physical bonding is primarily composed of friction (also known as the anchoring effect or mechanical interlocking) and van der Waals forces. The magnitude of friction is influenced by the roughness of the carbon fiber and matrix surfaces. Chemical bonding mainly involves the interactions of chemical bonds and hydrogen bonds. If the carbon fiber surface contains active functional groups, they can form chemical bonds or hydrogen bonds with the cement matrix, promoting a strong bond between the fiber and the matrix. The chemical reactions at the interface play a key role in interfacial bonding. As mentioned earlier, before reaching the peak load, the interfacial bonding strength of the fiber pull-out is mainly provided by chemical bonding. According to the FTIR test results, RCF has fewer active functional groups compared to VCF, which is why the interfacial bonding strength between RCF and the cement matrix is lower than that of VCF.

Chemical debonding energy reflects the energy required for the fiber to debond from the matrix when chemical interactions, such as chemical bonding or hydrogen bonding, occur at the fiber–matrix interface. VCF has a higher chemical debonding energy than recycled carbon fiber because the surface of virgin carbon fibers contains a large number of hydrophilic hydroxyl groups. As a result, VCF exhibits a stronger chemical bonding strength with the hydration products of the cement [53].

The difference in interfacial bonding friction strength between RCF and VCF is caused by the difference in surface roughness between them and the cement matrix. According to the atomic force microscopy (AFM) results, the surface roughness of RCF is significantly lower than that of VCF. This difference allows VCF to form a stronger mechanical interlocking with the cement matrix, which means that VCF must overcome a greater frictional force during the pull-out process, resulting in a higher interfacial friction bonding strength for VCF compared to RCF. Furthermore, pull-out energy is calculated from the area enclosed by the single-fiber pull-out load–displacement curve and its envelope. Since VCF has a higher pull-out load, its curve’s envelope area is larger, and thus, the pull-out energy is higher than that of RCF.

As discussed earlier, the surface modification of RCF by increasing surface roughness, enhancing the contact area at the interface, enriching the fiber surface morphology, and promoting physical anchoring, along with the addition of active functional groups, is crucial for achieving improved interfacial bond strength. The surface of RCF exhibits a disordered graphite structure, making it inert with few active sites. Therefore, increasing the number of active functional groups on the surface is essential for achieving better interfacial adhesion. Active functional groups not only significantly improve the fiber surface energy and roughness but also strengthen the chemical bonding between the fiber and the cement matrix, as well as the interfacial chemical debonding energy. This results in an increased energy barrier during fiber pull-out, thereby enhancing the mechanical properties of cement-based materials. Existing surface modification techniques for carbon fibers mainly include oxidation, surface coating, surface deposition, grafting, and impregnation treatments. Considering cost, production efficiency, and enhancement effects, and based on the relevant literature, the surface coating and grafting of chemical functional groups are considered effective methods. For example, coating RCF with silane coupling agents or grafting nanosilica onto the fibers can significantly improve the performance of RCF in practical construction applications. Nanosilica, with its high pozzolanic activity, reacts with the Ca (OH)_2_ that accumulates on the fiber surface during cement hydration, forming C-S-H gel, which densifies the interface. This greatly enhances the interfacial strength and chemical bonding energy.

**Figure 11:**
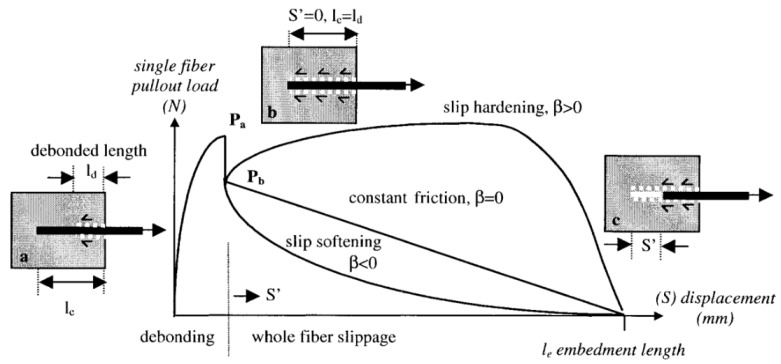
Typical single-fiber pull-out curve [17]. (**a**) A stable fiber debonding process occurs at the fiber/matrix interface. (**b**) The fiber is completely debonded. (**c**) Fiber slippage phase.

The slippage hardening coefficient is an important parameter that reflects the fiber reinforcement and toughening effect. It can be calculated from the initial slope of the load–displacement curve after the complete debonding of a single fiber. Figure 11 shows a typical single-fiber pull-out curve. Based on the different values of the slippage hardening characteristic coefficient *β*, the fiber slippage can be divided into three stages: slippage softening, slippage hardening, and constant friction. The corresponding values of the slippage hardening characteristic coefficient *β* are less than 0, greater than 0, and equal to 0, respectively. The slippage hardening characteristic coefficient *β* is calculated using Equation (6).

(6) $$\beta =\left(\frac{d}{L_{c}}\right)\left[\left(\frac{1}{\tau_{f}\pi d}\right){\left(\frac{\Delta P}{\Delta S^{\prime }}\right)}_{\left|S^{\prime }\to 0\right.}+1\right]$$

where *β* is the slippage hardening coefficient; Δ*P* is the small change in fiber pull-out force in N; Δ*S*′ is the small change in fiber pull-out displacement in mm; *S*’ is the displacement after the fiber has completely debonded in mm.

Table 4 shows the slippage hardening coefficient values for five groups of recycled carbon fibers (RCFs) and virgin carbon fibers (VCFs). Based on the results in the table, the average hardening coefficient for RCF is calculated to be 0.010, slightly lower than the 0.016 for VCF. This is mainly because the surface roughness of VCF is greater than that of RCF. During the sliding process, RCF experiences a stronger mechanical interlocking effect. Additionally, VCF contains a higher concentration of active functional groups compared to RCF, allowing VCF to form chemical bonds or hydrogen bonds with the matrix, thereby enhancing the interfacial interaction. As a result, the slip-hardening coefficient of VCF is slightly higher than that of RCF. As shown in Figure 9, after complete debonding (i.e., when the load sharply drops to point B), the fiber pull-out load increases during subsequent short-distance displacements, exhibiting a certain slippage hardening characteristic. Furthermore, although VCF has a stronger toughening effect than RCF, neither of the two carbon fibers shows significant slippage hardening effects due to their relatively smooth surfaces, with the slippage hardening coefficient *β* approaching 0. Studies have shown that the slippage hardening coefficient for PVA fibers ranges from 0.19 to 0.21 [54], which is much higher than that for RCF and VCF. PVA fibers, as a commercial fiber, can form strong hydrogen bonds with the hydration products in the cement matrix, significantly increasing the interfacial frictional resistance [51]. Moreover, as indicated by Equation (6), the slip-hardening coefficient is not only related to the surface chemical properties but also depends on the fiber diameter. The diameter of PVA fibers is typically around 40 μm, which is much larger than that of RCF and VCF, which is 7 μm. Therefore, for fibers of the same length, PVA fibers will exhibit a higher slip-hardening coefficient. Therefore, RCF-reinforced cementitious materials are less likely to experience multiple crack formations and failures, unlike PVA fibers. Surface modification techniques such as oxidation, coating, or grafting functional groups can enhance the interfacial bonding between RCF and the cement matrix, thus enabling RCF to potentially achieve a reinforcing effect comparable to that of commercial fibers.

## 4. RCF Strengthens the Bridging Stress of Cementitious Materials

The results above show that the addition of RCF can improve the tensile strength of cementitious materials. This is because, when cracks form in the cementitious materials, RCF generates bridging stress at the crack sites. This stress not only ensures that the cementitious materials maintain considerable load-bearing capacity even after crack propagation but also effectively suppresses the continuous expansion of the cracks, thereby enhancing the overall performance of the cementitious materials. To further understand the mechanism of RCF’s reinforcement effect on cementitious materials, the bridging stress of RCF in the cement matrix is analyzed.

### 4.1. Establishment of a Single-Fiber Pull-Out Model

**Figure 12:**
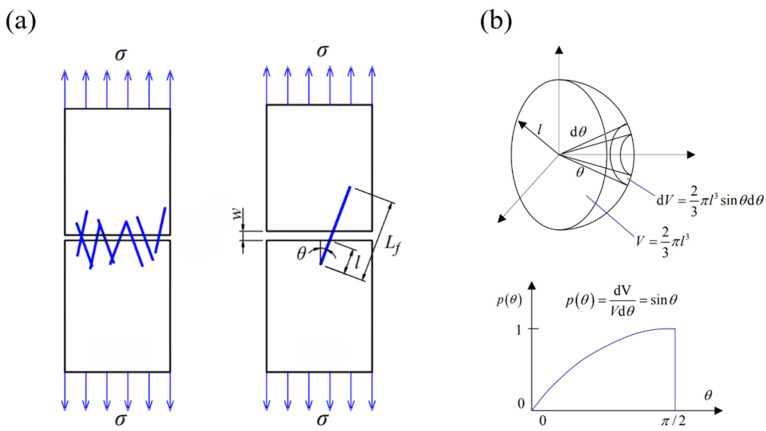
(**a**) Schematic diagram of fiber cementitious material cracking; (**b**) schematic diagram of probability function of fiber embedment angle distribution.

The fibers added to cementitious materials are randomly distributed in various orientations. Therefore, when establishing the fiber-bridging stress model, it is necessary to consider the random distribution function of the fibers. Figure 12a shows a schematic of fiber-reinforced cement-based material cracking. As shown in the figure, when the matrix cracks, the fibers randomly distributed in the matrix intersect with the crack surface at a normal angle. The length of the fiber embedded on the shorter side of the crack is denoted as *l*, and the crack width is denoted as *w*. When fibers of fixed length *L_f_* are used and are uniformly and randomly distributed in the cement matrix in three-dimensional space, the fibers are evenly distributed on both sides of the crack. The distribution probability function of the embedded fiber length can be calculated using Equation (7).

(7) $$P\left(l\right)=2/L_{f}$$

where *L_f_* is the total length of the fiber. From Equation (7), it can be seen that the embedded fiber length *l* is not affected by the normal angle between the fiber and the crack surface; they are two independent, uncorrelated variables.

The distribution of the angle between the fibers and the normal angle to the crack surface can be equivalently represented as the distribution of fiber embedment points on a hemisphere, as shown in Figure 12b. Since the fibers are randomly and uniformly distributed within the matrix, the number of fibers within the conical volume is proportional to the matrix volume and can be calculated using Equations (8) and (9).

(8) $$N\left(\theta \right)=V_{f}\cdot dV$$

(9) $$dV=\left(2\pi l\sin\theta \right)\cdot \left(ld\theta \right)\cdot \frac{l}{3}$$

where *N*(*θ*) is the number of fibers distributed within the conical volume, and *V_f_* is the fiber volume fraction. The total number of fibers falling within the entire hemisphere is calculated using Equation (10).

(10) $$N_{all}=V_{f}\cdot \frac{2}{3}\pi l^{3}$$

Therefore, the distribution probability function of the fiber embedment angle is given by Equation (11).

(11) $$P\left(\theta \right)=\frac{dV}{Vd\theta }=\sin\left(\theta \right)$$

To simplify the analysis, the following basic assumptions are made when studying the bridging stress model of recycled carbon fibers: (1) All fibers are short-cut fibers with fixed lengths, randomly distributed in various orientations within the cement matrix. Their length distribution function and angle distribution function are represented by the derived Equations (7) and (11), respectively. (2) During crack propagation, all fibers at the crack sites are pulled out rather than broken. (3) After the cracking of the specimen, all cracks converge into a main crack, which is perpendicular to the direction of the applied force. (4) According to the study by Leung and Ybanez [55] on the influence of the peak load of the fiber pull-out curve with the change in burial angle, when fibers are pulled out at an inclined angle, their peak load is *e^fθ^* times that observed during vertical pull-out [56]. (5) The fiber bridging efficiency *η_B_* is defined to represent the relationship between the number of fibers bridging the crack and the orientation effect. When fibers are distributed in three-dimensional space, this value is 0.5 [57]. (6) Since the volume fractions of RCF and VCF in this study are very low, the interactions between fibers and the local debonding loss of the matrix are neglected. Therefore, the fiber volume fraction influence coefficient *ρ*(*Vf*) = 1. In the above assumptions, *f* refers to the angle friction coefficient, also known as the cushioning coefficient. This coefficient represents the amplification effect caused by the inclined embedding of fibers and is dependent on the material properties. It is typically determined empirically. Relevant studies indicate that for synthetic fibers, the angle friction coefficient for PE fibers ranges from 0.2 to 0.5 [58,59]. Since RCF is a synthetic fiber, in this study, a value of *f* = 0.2 is adopted based on related research.

Since RCF is a synthetic fiber, and considering the convenience of applying the theoretical model, this study adopts the synthetic fiber pull-out model 1 proposed by Du et al. [60], which does not require consideration of the descending segment parameters of the fiber pull-out load curve. This model is used to investigate the bridging stress of recycled carbon fibers (RCFs), analyze the reinforcement mechanism of RCFs, and explore the differences in their enhancement effect compared to virgin carbon fibers. The pull-out model, considering both the embedment length *l* and embedment angle *θ*, is given by Equation (12).

(12) $$P_{f}\left(l,\theta ,u\right)=\left\{\begin{array}{l} k_{0}\cos\theta u\;0<u\leq L_{1},\;0<\theta <\frac{\pi }{2}\\ k_{1}\left(u-l\right){\;L}_{1}<u<l,\;0<\theta <\frac{\pi }{2}\\ 0,\;l=0\;\mathrm{or}\;u=0\;\mathrm{or}\;\theta =0\;\mathrm{or}\;u>l \end{array}\right.$$

where *u* is the displacement at which the fiber is pulled out according to the theoretical model of the pull-out curve. *L_1_* is the displacement corresponding to the peak load in the theoretical model of the pull-out curve, $L_{1}=\frac{l}{L_{c}}\cdot \frac{P_{0}e^{f\theta }}{k_{0}\cos\theta }$; *l* is the embedment length of the short fiber segment in the theoretical pull-out model. *P_0_* is the peak load in the single-fiber pull-out test when the embedment angle is 0, and the corresponding fiber embedment length is *L_c_*. *k*_0_ is the slope of the ascending segment of the pull-out curve, and its value is independent of the fiber embedment angle and embedment length. *k*_1_ is the slope of the descending segment of the pull-out curve, $k_{1}=\frac{P_{0}e^{f\theta }}{P_{0}e^{f\theta }/k_{0}\cos\theta -L_{c}}$, and it is independent of the fiber embedment length. Since the range of fiber embedment length *l* is [0,$\frac{L_{f}}{2}$], and the range of embedment angle *θ* is [0,$\frac{\pi }{2}$], Equation (12) is applicable to the micro pull-out model of recycled carbon fibers with different embedment lengths and angles.

Once the single-fiber pull-out model and the distribution pattern of the fibers within the matrix are obtained, and the above assumptions are satisfied, the bridging stress of the recycled carbon fiber cementitious materials can be calculated using the superposition principle, as shown in Equation (13) [61].

(13) $$\sigma_{fB}\left(u\right)=\eta_{B}\rho \left(V_{f}\right)\frac{V_{f}}{A_{f}}\int_{0}^{\frac{2}{L_{f}}}\int_{0}^{\frac{\pi }{2}}P_{f}\left(l,\theta ,u\right)\frac{2}{L_{f}}\sin\theta d\theta dl$$

where *A_f_* is the cross-sectional area of a single fiber, $A_{f}=\frac{\pi d^{2}}{4}$. When calculating the fiber-bridging stress using the theoretical model, several key parameters need to be determined. Among them, *V_f_*, *A_f_*, *L_f_*, and *f* are intrinsic material properties and can be directly obtained. *P*_0_ and *k*_0_ are determined through single-fiber pull-out tests. Once all the key parameters are obtained, the theoretical model equations can be implemented through programming and used in numerical simulation software Wolfram Mathematica v14.0 to calculate the fiber-bridging stress curve.

### 4.2. Calculation Result

**Figure 13:**
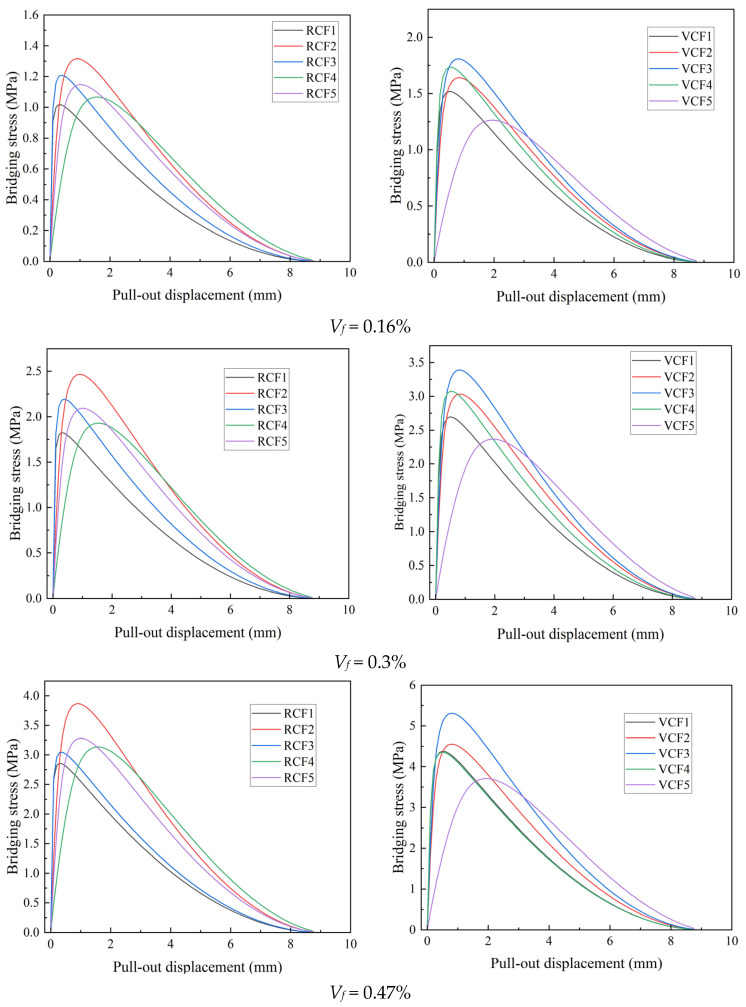
Theoretical bridging stress curves of fibers at different dosages.

Figure 13 shows the fiber-bridging stress curves calculated using the theoretical model for different fiber dosages, with five data points for each parameter set. As shown in the figure, the bridging stress increases with the fiber dosage. Additionally, for the same fiber dosage, the bridging stress of VCF is higher than that of RCF. According to Equation (13), the theoretical bridging stress is primarily influenced by the peak pull-out load *P*_0_ and the slope *k*_0_ of the ascending segment of the pull-out curve. The larger the values of *P*_0_ and *k*_0_, the higher the theoretical bridging stress. From the results of the single-fiber pull-out tests, it is evident that the *P*_0_ and *k*_0_ values for VCF are greater than those for RCF, meaning that VCF exhibits a higher theoretical bridging stress than RCF. Moreover, according to Equation (13), when parameters such as *A_f_*, *L_f_*, and *f* are the same, the theoretical bridging stress is determined by the fiber volume fraction *V_f_*. As the fiber dosage increases, more fibers are distributed uniformly within the cement matrix, leading to a greater contribution of fibers in bridging the cracks in the matrix, thus enhancing the toughness. The bridging stress shown in Figure 13 is positively correlated with the tensile strength in Figure 5, meaning that the greater the bridging stress provided by the fibers, the higher the tensile strength of the cement-based material. The trends in both cases are consistent with changes in fiber content. The tensile test results validate the relationship between bridging stress and fiber content variation from the perspective of macro-mechanical performance.

Ma et al. [62] proposed an equation for calculating the fiber-bridging stress based on the interfacial friction bonding strength obtained from single-fiber pull-out tests. The calculation equation is shown in Equation (14). For simplicity, the formula neglects fiber fracture, slippage hardening, and buffering effects.

(14) $$\sigma_{\mathrm{fB}}=\frac{4V_{f}\tau_{f}}{L_{f}d}{\left(\frac{L_{f}}{2}\right)}^{2}\cdot \eta_{Β}$$

**Figure 14:**
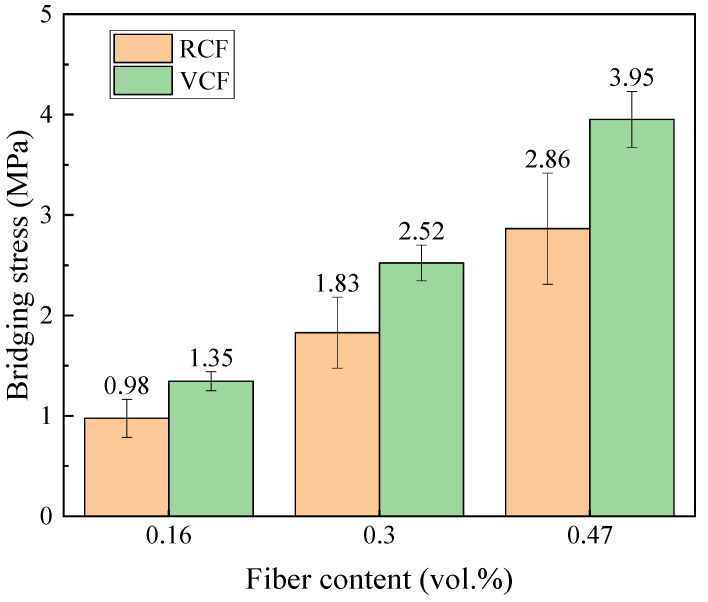
Fiber bridge stress test values.

In this study, the fiber-bridging stress calculated using Equation (14) is referred to as the experimental bridging stress value and is summarized in Figure 14. As shown in the figure, in accordance with the trend of the theoretical bridging stress values, the experimental bridging stress values also increase with the increase in fiber content. From Equation (14), it can be seen that the fiber-bridging stress values are primarily influenced by the interfacial friction bonding strength, which is directly related to the interfacial roughness and compactness between the fiber and the cement matrix [63]. Since the interfacial friction bonding strength between virgin carbon fibers and the cement matrix is higher than that of recycled carbon fibers, the experimental bridging stress value for cementitious materials reinforced with virgin carbon fibers is greater than that for materials reinforced with recycled carbon fibers, assuming the same fiber content.

To verify the accuracy of the fiber-bridging stress model used for recycled carbon fibers in this study, the error between the theoretical average bridging stress $\sigma_{fB}^{T}$ and the experimental average bridging stress $\sigma_{fB}^{E}$ for cementitious materials reinforced with recycled and virgin carbon fibers at different fiber contents was calculated to analyze the degree of approximation between these two values. The corresponding results are summarized in Table 5. From Table 5, it can be seen that the mean error between the theoretical and experimental bridging stress for RCF ranges from 13.3% to 17.3%, while for VCF, the mean error ranges from 12.9% to 17.7%. Furthermore, as the fiber content increases, the mean error between the theoretical and experimental bridging stress decreases gradually. The model’s prediction of bridging stress provides a theoretical reference for the engineering application of RCF-reinforced cementitious materials.

## 5. Conclusions

This study characterizes the performance of RCF and investigates the interfacial bonding properties between RCF and the cement matrix, providing a theoretical foundation for future surface modification treatments aimed at enhancing the overall performance of RCF. The following conclusions were drawn from this study:

(1) The interfacial bonding strength, interfacial friction bonding strength, chemical debonding energy, and pull-out energy of RCF are, respectively, 43.1%, 38.3%, 105.6%, and 82.4% lower than those of VCF. Additionally, based on the single-fiber pull-out curve and slippage hardening coefficient *β*, RCF does not exhibit a significant slippage hardening effect within the cement matrix.

(2) The fiber pull-out model can accurately calculate the bridging stress provided by RCF in cementitious materials, and the bridging stress increases with the increase in RCF content. According to the model calculations, when the RCF volume fraction is 0.16%, the bridging stress is 1.25 MPa. When the RCF volume fraction increases to 0.47%, the bridging stress rises to 3.4 MPa.

(3) RCF can significantly improve the tensile properties of cementitious materials, and this improvement increases with the fiber content, with a 28-day tensile strength increase of 8.8% to 35.48%. The tensile test results validate the pattern of bridging stress variation with fiber content from a macroscopic mechanical perspective.

## Figures and Tables

**Table 1:** Physical properties of 42.5 ordinary silicate cement.

Heat Loss (%)	Specific Surface Area (m^2^/kg)	Initial Setting Time (min)	28-Day Flexural Strength (MPa)	28-Day Compressive Strength (MPa)
3.62	354	216	8.6	52.6

**Table 2:** VCF and RCF performance parameters.

	Length (mm)	Diameter (μm)	Carbon Content (%)	Density (g·cm^−3^)	Tensile Strength (MPa)	Tensile Modulus (GPa)
VCF	18	7	96	1.8	3450	230
RCF	18	7.28	92.8	1.79	2866	230

**Table 3:** Specimen number and mix proportion.

	Cement	Water	Sand	Carbon Fiber (vol.%)	Water Reducer (g)	Flowability (mm)
CF0	1	0.5	1.5	0	0.5	250
RCF0.16	1	0.5	1.5	0.16	0.5	232.5
RCF0.30	1	0.5	1.5	030	0.5	214
RCF0.47	1	0.5	1.5	0.47	0.5	203
VCF0.16	1	0.5	1.5	0.16	0.5	222.5
VCF0.30	1	0.5	1.5	0.30	0.5	209
VCF0.47	1	0.5	1.5	0.47	0.5	190

**Table 4:** Slippage hardening coefficient values of different carbon fibers with cementitious matrix.

	Specimen Number				
	1	2	3	4	5
RCF	0.015	0.007	0.007	0.015	0.006
VCF	0.014	0.032	0.004	0.014	0.017

**Table 5:** Error analysis of the average theoretical bridge stress and the average test bridge stress under different fiber dosages.

	$\sigma_{fB,RCF}^{T}$ (MPa)	$\sigma_{fB,RCF}^{E}$ (MPa)	RCF Error Mean	$\sigma_{fB,VCF}^{T}$ (MPa)	$\sigma_{fB,VCF}^{E}$	VCF Error Mean
*V_f_* = 0.16%	1.15	0.98	17.3%	1.59	1.35	17.7%
*V_f_* = 0.3%	2.10	1.83	14.8%	2.91	2.52	15.5%
*V_f_* = 0.47%	3.24	2.86	13.3%	4.46	3.95	12.9%

## Data Availability

The original contributions presented in this study are included in the article. Further inquiries can be directed to the corresponding author.
